# Intravenous thrombolysis with alteplase in the treatment of acute cerebral infarction

**DOI:** 10.12669/pjms.38.3.4521

**Published:** 2022

**Authors:** Lilin Gao, Shaojie Zhang, Xuewen Wo, Xiangpeng Shen, Qiangyuan Tian, Guoqing Wang

**Affiliations:** 1 Lilin Gao, Department of Neurology, Binzhou People’s Hospital, Shandong 256610, China; 2 Shaojie Zhang, Department of Neurology, Binzhou People’s Hospital, Shandong 256610, China; 3 Xuewen Wo, Department of Neurology, Binzhou People’s Hospital, Shandong 256610, China; 4 Xiangpeng Shen, Department of Neurology, Binzhou People’s Hospital, Shandong 256610, China; 5 Qiangyuan Tian, Department of Neurology, Binzhou People’s Hospital, Shandong 256610, China; 6 Guoqing Wang, Department of Neurology, Binzhou People’s Hospital, Shandong 256610, China

**Keywords:** Alteplase, Urokinase, Acute cerebral infarction, Monocyte chemoattractant protein-1

## Abstract

**Objectives:**

To compare the efficacy and safety of intravenous thrombolysis with alteplase and intravenous thrombolysis with urokinase for patients with acute cerebral infarction.

**Methods:**

This prospective study included 140 patients with acute cerebral infarction who were admitted to our hospital between June 2018 and June 2019. They were randomly divided into two groups. The control group (70 cases) was treated with urokinase intravenous thrombolysis, and the observation group (70 cases) was given alteplase intravenous thrombolytic therapy. The treatment efficacy and safety of the two groups were compared.

**Results:**

The total effective rate of the observation group was 95.7%, and that of the control group was 78.6%, i.e., the total effective rate of the observation group was significantly superior to the that of the control group (P < 0.05). After treatment, the observation group had significantly lower National Institutes of Health Stroke Scale (NIHSS) score and significantly higher mini-mental state examination (MMSE) score than the control group; the difference was statistically significant (P<0.05). After treatment, the levels of inflammatory factors of both groups significantly decreased compared to before treatment, and the decrease in the observation group was larger than that in the control group (P<0.05). The levels of serum homocysteine (Hcy) and monocyte chemoattractant protein-1 (MCP-1) in the observation group were significantly lower than those in the control group after treatment, and the differences were statistically significant (P<0.05). The incidence of hemorrhagic adverse reaction in the observation group was lower than that in the control group (P<0.05).

**Conclusion:**

In the treatment of acute cerebral infarction, ccompared with urokinase, alteplase can further relieve cognitive impairment and promote the recovery of nerve function through inhibiting levels of inflammatory factors and levels of serum Hcy and MCP-1.

## INTRODUCTION

Stroke is famous for its high mortality, high disability rate, high recurrence rate, and low cure rate. At present, stroke has become the second leading cause of death in the world. Among 1.4 billion people in China, the number of new cases of stroke is about 2.5 million every year, and the annual average number of deaths is nearly 1.6 million, i.e., 157 people dies of stroke in every 100000 patients, which makes stroke surpass heart disease and become the leading cause of death in China. Acute ischemic cerebral infarction is the most common type of stroke, accounting for 43% - 79% of all stroke.^1^,^2^ Epidemiological surveys show that the incidence of ischemic stroke in China has begun to be higher among youth in recent years with the development of social economy and the change of people’s lifestyle. Therefore, to find positive and effective specific treatment methods has become the focus of clinical work and scientific research in various medical units.^3^,^4^

The emergence of ultra early intravenous thrombolytic therapy technology has achieved a new breakthrough in the effective treatment of acute ischemic cerebral infarction.^5^ With the development of clinical medicine, the types of thrombolytic drugs are also increasing, and there are some differences in the efficacy of different drugs.^6^ Alteplase is the representative of the second generation of thrombolytic drugs, which can combine with fibrin and selectively activate plasminogen binding with fibrin to transform it into plasminogen but will not activate plasminogen in the circulatory system.

Recombinant tissue plasminogen activator (rt-PA) has a half-life of only four to six minutes, and it selectively activates plasminogen. It has a weak systemic effect on the components in the whole circulatory system and blood system and is not easy to cause bleeding. It has the advantages of strong thrombolytic ability, high specificity, and good safety. Many researchers in China and abroad have reported the curative effect of rt-PA, but there are some differences in the results of the relevant studies. In 2008, a study published by Hacke et al. has confirmed that intravenous thrombolytic therapy with rt-PA within 3-4.5 hours after onset can effectively relieve symptoms, which provides an opportunity for those patients with acute ischemic cerebral infarction who cannot receive thrombolytic therapy within three hours after onset due to various reasons.^7^ In 2010, Kennedy RL et al. found that intravenous thrombolytic therapy with rt-PA within four and a half hours after onset could still benefit, but the risk of thrombolytic therapy for patients with more than four and a half hours of onset was far greater than the benefit.^8^

A study on rt-PA intravenous thrombolytic therapy in Asian population made by Sharma VK et al. showed that rt-PA intravenous thrombolytic therapy remained to be effective in reducing the long-term mortality or disability rate in patients with acute ischemic cerebral infarction whose onset time was shorter than six hours.^9^ Although the above studies have confirmed the effectiveness of alteplase, few studies compare its efficacy with other drugs. Therefore, 140 patients with acute cerebral infarction in our hospital were selected by this study, and the efficacy and safety of alteplase and urokinase intravenous thrombolysis in the treatment of acute cerebral infarction were compared and analyzed. The report is as follows.

## METHODS

This study is prospective. One hundred and forty patients with acute cerebral infarction who were admitted to our hospital between June 2018 and June 2019 were selected as the research subjects, and they were divided into two groups according to the random number method, with 70 cases in each group. There were 42 males and 28 females in the control group; the age of the patients was 38-76 years old, with an average age of (60.12±4.21) years; the time from onset to treatment was 2 h-5 h (average (3.12±0.23) hour). There were 38 males and 32 females in the observation group; the age of the patients was 35-78 years (average (65.12±4.67) years); the time from onset to treatment was one to five hours average (3.33±0.43) hours). There was no difference in the basic data between the two groups (P>0.05); thus, the results were comparable. This study was approved (Ethical Approval Form No. 219, Dated October 10^th^ 2020) by the medical ethics committee of our hospital. All patients and their families were informed of the study content and had signed the consent form.

### Inclusion and exclusione criteria

Inclusion criteria included meeting the diagnostic criteria for acute cerebral infarction in Chinese Guidelines for Diagnosis and Treatment of Acute Ischemic Stroke 2018;^10^ being diagnosed as acute cerebral infarction by head computed tomography (CT) and magnetic resonance imaging (MRI), and satisfying the requirements of intravenous thrombolysis described in Scientific Statement of Intravenous Thrombolysis of Acute Ischemic Stroke by Chinese Stroke Association.^11^ Patients who had cardiogenic cerebral infarction, hemorrhagic cerebral infarction, contraindications to thrombolytic therapy, infections, other serious diseases, hepatic and renal dysfunction, uncontrollable hypertension and diabetes were excluded.

After admission, the two groups were given the conventional treatment such as fluid infusion, oxygen inhalation, intracranial pressure reduction, electrolyte disorder correction, and anti-platelet aggregation according to the disease condition of the patients after being admitted to the hospital. The control group was treated with urokinase (Wuhan Humanwell Pharmaceutical Co., Ltd., SFDA approval number H42021790) intravenous thrombolysis, with a dose of 20000-40000 U/kg, and the maximum one-time dose was no more than 1.5 million units. Urokinase was mixed with 100 mL of 0.9% NaCl injection, and the injection was intravenously dripped within 30 minutes. The observation group was given alteplase (Germany Boehringer-Ingelheim Pharmaceutical Factory, SFDA approval number S20110052) intravenous thrombolysis at the dose of 0.9 mg/kg, for twice. Firstly, 10% was intravenously injected within 10 s, and the remaining dose was dissolved into the special mixture and intravenous dripped within 90 min, once a day. After thrombolytic therapy, activated partial thromboplastin time (APTT) was dynamically monitored to adjust the dosage. Moreover, corresponding symptomatic treatment was given according to the patient’s condition. The treatment time was 10 days in both groups.

### Detection methods

Firstly, 3 ml of fasting venous blood was taken from the patient and centrifuged for 12 minutes at 3000 r/min to separate serum. (1) Serum tumor necrosis factor-α (TNF-α), interleukin-6 (IL-6), and monocyte chemoattractant protein-1 (MCP-1) were detected by the enzyme-linked immunosorbent assay (ELISA) using a kit (Shanghai Ruifan Biotechnology Co., Ltd., China). (2) The level of serum homocysteine (Hcy) was detected by the fluorescence polarization immunoassay using a kit (Xiamen Huijia Biotechnology Co., Ltd., China). (3) The level of serum high sensitivity C-reactive protein (hs-CRP) was detected by the immunoenhancement turbidimetry using a kit (Guangzhou Weijia Technology Co., Ltd., China), and the operation strictly followed the instructions of the kit.

### Observation index

Clinical effect: the clinical effects of the two groups were observedDegree of nervous functional defects and cognitive function: The degree of nervous functional defects and cognitive function were evaluated by National Institutes of Health Stroke Scale (NIHSS)^12^ and Mini-Mental State Examination (MMSE) before and after treatment; the total score of NIHSS was 0~42 points, and the score was positively correlated with the degree of nervous functional defects; the total score of MMSE was 0~30 points, and the score was in direct proportion to the cognitive function. Serum inflammatory cytokines: the serum levels of TNF-α, IL-6, hs-CRP, Hcy, and MCP-1 before and after treatment were observed. Adverse reactions: the incidence of intracranial bleeding, gingival bleeding, and gastrointestinal bleeding was observed.

### Evaluation criteria of efficacy

The efficacy of the two groups was evaluated based on the changes of the NIHSS score and the improvement of clinical symptoms.^13^ The treatment was evaluated as significantly effective if living abilities such as language and activity has recovered to be normal and the NIHSS score reduced more than 50% after treatment, as effective if living abilities such as language and activity has improved and the NIHSS score dropped 20% ~ 50%, and as ineffective if living abilities such as language and activity had no improvement and the NIHSS score reduced less than 20%. The calculation formula of the total effective rate = the percentage of significantly effective cases + the percentage of effective cases.

### Statistical analysis

Professional treatment was carried out by SPSS 23.0. The measurement data were expressed as Mean±SD, and t-test was used. The enumeration data such as the total effective rate were expressed in the form of percentage (%), and the Chi-square test was used. If P>0.05 for the difference, then it meant that there was a statistical significance.

## RESULTS

### Comparison of clinical treatment effect between the two groups

The total effective rate of the observation group was 95.7%, and that of the control group was 78.6%. The total effective rate of the observation group was significantly superior to that of the control group, and the difference had statistical significance (P<0.05, Table-I).

**Table-I:** Comparison of clinical treatment effect between the two groups (%).

Group	Significantly effective	Effective	Ineffective	Total effective rate
Observation group	42(60.0%)	25(35.7%)	3(4.3%)	67(95.7%)
Control group	22 (31.4%)	33 (47.1%)	15 (21.4%)	55 (78.6%)
X^2^				9.716
P				<0.05

### Comparison of NIHSS and MMSE scores between the two groups before and after treatment

There was no significant difference in NIHSS and MMSE scores between the two groups before treatment (P>0.05). NIHSS scores of the two groups after treatment were lower than those before treatment. The MMSE score of both groups improved after treatment, and the MMSE score of the observation group was significantly higher than that of the control group (P<0.05, Table-II).

**Table-II:** NIHSS and MMSE scores between the two groups (Mean±SD).

Group	NIHSS score		MMSE score	

	Before treatment	After treatment	Before treatment	After treatment
Observation group	29.12±2.31	16.63±1.72	14.83±3.08	25.60±4.13
Control group	27.75±2.52	20.09±2.11	15.26±2.72	21.46±3.67
t	0.683	8.946	0.746	5.473
P	0.496	<0.01	0.459	<0.01

### Comparison of serum inflammatory cytokines between the two groups before and after treatment

The levels of serum TNF-α, IL-6, and hs-CRP of the two groups had no significant differences before treatment (P>0.05). After treatment, the levels of serum TNF-α, IL-6, and hs-CRP decreased in both groups, and the levels in the observation group were significantly lower than those in the control group (P<0.05, Table-III).

**Table-III:** Serum TNF-α, IL-6, and hs-CRP levels between the two groups (Mean±SD).

Time	Group	TNF-α (pg/mL)	IL-6 (pg/mL)	hs-CRP (mg/L)
Before treatment	Observation group	23.85±5.62	24.35±4.61	14.26±2.73
	Control group	22.98±5.38	23.43±4.16	14.49±3.11
	t	0.834	1.297	0.384
	P	0.412	0.276	0.702
After treatment	Observation group	10.76±2.41	11.52±2.28	6.03±1.87
	Control group	13.18±2.81	15.80±3.06	8.74±2.24
	t	4.783	8.172	6.710
	P	<0.01	<0.01	<0.01

### Comparison of serum Hcy and MCP-1 levels between the two groups before and after treatment

The serum Hcy and MCP-1 levels of the two groups had no significant differences before treatment (P>0.05). After treatment, the serum Hcy and MCP-1 levels of the two groups decreased after treatment, and the levels in the observation group were significantly lower than those in the control group (P<0.05, Table-IV).

**Table-IV:** Serum Hcy and MCP-1 levels between the two groups.

Group	Hcy (μmol/L)		MCP-1 (pg/mL)	

	Before treatment	After treatment	Before treatment	After treatment
Observation group	32.48±4.28	16.42±2.16	149.15±18.26	92.58±8.28
Control group	31.62±4.76	20.84±3.05	146.95±20.49	116.96±12.42
t	0.968	8.594	0.587	11.837
P	0.226	<0.01	0.562	<0.01

### Comparison of incidence of hemorrhagic adverse reaction between the two groups after treatment

The bleeding incidence of the observation group and control group was 11.4% and 32.9%, respectively, i.e., the incidence of hemorrhagic adverse reaction of the observation group was significantly lower than that of the control group (P<0.05, Table-V).

**Table-V:** Incidence of hemorrhagic adverse reaction between the two groups.

Group	Intracranial hemorrhage	Gingival bleeding	Gastrointestinal bleeding	Total incidence of adverse reactions
Observation	4 (5.7%)	0 (0.0%)	4 (5.7%)	8 (11.4%)
Control group	9 (12.9%)	9 (12.9%)	5 (7.1%)	23 (32.9%)
X^2^	-	-	-	6.842
P	-	-	-	<0.05

## DISCUSSION

In the clinical treatment of acute cerebral infarction, thrombolysis and dredging is the main treatment method. Due to the simple operation and low price, intravenous thrombolysis is generally used in clinical treatment.^14^ Urokinase is a commonly used drug in thrombolysis. Urokinase is mainly extracted from the urine of healthy people or cultured in human kidney tissue, which is an enzyme protein.^15^ It can play a direct role in the endogenous fibrinolysis system, i.e., catalyzing the schizolytic plasminogen into plasmin to degrade fibrin clot.^16^ However, the half-life of urokinase is relatively short, and the antigenicity does not exist. Although it plays a direct role in thrombolysis, it is only aimed at the newly formed thrombus. For some old thrombus, the action degree is low. Therefore, from the perspective of clinical treatment practice, it still needs to be improved.^17^ Alteplase, as a second-generation thrombolytic drug, is also a specific fibrinolytic agent. It can selectively activate fibrinolytic enzyme in thrombus site and convert it to plasmin, which is featured by fast thrombolysis and good efficacy.^18^,^19^ The results showed that the total effective rate of the observation group treated with alteplase was significantly higher than that of the control group treated with urokinase, and the improvement of the NIHSS score and MMSE score in the observation group after treatment was significantly superior to that in the control group (P<0.05). The above results showed that the efficacy of intravenous thrombolysis with alteplase was better than that of urokinase. Some similar research results support the view of this paper.^20^ For example, in Liu’s study,^21^ 98 patients were selected and treated respectively. The effective rate of patients who were treated with alteplase was 95.92%, which was far higher than that of patients who were treated with urokinase (81.63%). The same is true for the neurological function score. The above results demonstrated that this research had representative significance.

A study has verified that inflammatory response is one of the important pathophysiological mechanisms of acute ischemic stroke,^22^ and TNF-α, IL-6, and hs-CRP are important inflammatory mediators of secondary brain injuries in patients with acute ischemic stroke. This study analyzed the serum levels of inflammatory factors before and after treatment. The levels of serum TNF-α, IL-6, and hs-CRP in the two groups after treatment were lower than those before treatment, suggesting that rt-PA thrombolysis and urokinase intravenous thrombolysis could reduce the levels of serum inflammatory factors. The further comparison of serum inflammatory factor levels between the two groups after treatment showed that the levels of TNF-α, IL-6, and hs-CRP in the observation group were lower than those in the control group after treatment, indicating that rt-PA thrombolytic therapy was more effective in expanding the blood vessels at the infarct site, accelerating the recovery of blood flow, inhibiting calcium overload, removing free radicals, blocking the release of inflammatory factors, such as TNF-α, IL-6 and hs-CRP, reducing the body’s inflammatory reaction, and alleviating the inflammatory damage of inflammatory mediators to brain cells, and promoting the recovery of nerve function. When cerebral ischemic injury occurs, neurons, macrophages and endothelial cells will secrete a large amount of MCP-1 and up regulate the expression of adhesion molecules under the induction of free radicals, thus affecting the blood supply of brain tissues and further aggravating the hypoxic-ischemic injury of brain tissues. The correlation analysis made by Ge et al.^23^ showed that serum MCP-1 level was positively correlated with the area and NIHSS score of acute ischemic stroke, which can be used as an effective index for clinical diagnosis and prognosis evaluation of acute ischemic stroke. Hcy can damage vascular endothelial cells, abnormally proliferate vascular smooth muscle cells, induce platelet aggregation, change blood composition, promote thromboxane A2 production, increase the risk of thrombosis, accelerate calcium and lipid deposition, and promote the occurrence and progress of acute ischemic stroke. Li et al. found that the serum Hcy level increased significantly with the increase of the volume of cerebral infarction lesions and the aggravation of neurological deficit in patients with acute ischemic stroke.^24^ The data of this study suggested that alteplase combined with rt-PA intravenous thrombolytic therapy could significantly reduce the serum Hcy and MCP-1 levels in patients with acute ischemic stroke, which may be an important means to regulate the neurological impairment in patients with acute ischemic stroke, but the specific mechanism needs to be further explored.

In addition, the results of this study showed a lower incidence rate of bleeding adverse reactions after alteplase treatment compared to urokinase treatment. The reason might be that alteplase avoided the disadvantage of urokinase, i.e., dissolving fibrous protein and activating plasminogen, thereby promoting the thrombolytic effect and greatly reducing the incidence of bleeding complications, which is in accordance with the results of a previous study.^25^

### Limitations of the study

It includes small sample size, which limited the generalization of the findings and led to possible bias in the data results. Further verification will be carried out by expanding the sample size in future studies.

## CONCLUSION

In conclusion, within the effective time, alteplase intravenous thrombolysis has a higher cure rate than urokinase and a significantly lower incidence of bleeding in the treatment of for acute cerebral infarction. Alteplase intravenous thrombolysis can effectively inhibit inflammatory factor levels, reduce serum Hcy and MCP-1 levels, further relieve cognitive dysfunction and promote neurological recovery, which is worth further clinical promotion and application.

### Authors’ Contribution:

**LLG, SJZ & XWW:** Study design, data collection and analysis.

**SJZ, XWW, XPS & QYT:** Manuscript preparation, drafting, revising and is responsible for integrity of the study.

**LLG & GQW:** Review and final approval of manuscript.
